# Demystifying penetrating atherosclerotic ulcer of aorta: unrealised tyrant of senile aortic changes

**DOI:** 10.34172/jcvtr.2021.15

**Published:** 2021-01-30

**Authors:** Rahul Dev, Khorwal Gitanjali, Darbari Anshuman

**Affiliations:** ^1^Department of Radiodiagnosis and Imaging, All India Institute of Medical Sciences, Rishikesh, Uttarakhand-249203, India; ^2^Department of Anatomy, All India Institute of Medical Sciences, Rishikesh, Uttarakhand-249203, India; ^3^Department of Cardiothoracic and Vascular Surgery (CTVS), All India Institute of Medical Sciences, Rishikesh, Uttarakhand-249203, India

**Keywords:** Aortic Dissection, Computed Tomography Angiography, Intramural Hematoma, Thoracic Endovascular Aortic Repair, Penetrating Atherosclerotic Ulcer

## Abstract

This review article describes demographic features, comorbidities, clinical and imaging findings, prognosis, and treatment strategies in penetrating atherosclerotic ulcer (PAU) and closely related entities using google scholar web search. PAU is one of the manifestations of the acute aortic syndrome (AAS) spectrum. The underlying aorta invariably shows atherosclerotic changes or aneurysmal dilatation. Hypertension is the most common contributing factor, with chest or back pain being the usual manifestation. Intramural hematoma (IMH) is the second entity associated with both PAU and aortic dissection (AD), more so with the latter. Chest radiograph can show mediastinal widening, pleural, or pericardial fluid in rupture. Computed tomography angiography (CTA) is the imaging modality of choice to visualize PAU, with magnetic resonance imaging (MRI) and transoesophageal echocardiography (TEE) adding diagnostic value. Lesser-known entities of intramural blood pool (IBP), limited intimal tears (LITs), and focal intimal disruptions (FID) are also encountered. PAU can form fistulous communication with adjacent organs whereas IMH may propagate to dissection. CTA aids in defining the management, open or endovascular options in surgical candidates.

## Introduction

Penetrating atherosclerotic ulcer (PAU) of the aorta was described by Shennan in 1934 with Vilacosta, and Roman coined the term acute aortic syndrome in 2001.^1^ Recently, terms like incomplete dissection and intimomedial flap are introduced.^2,3^ PAU targets aortic intima with loss of integrity manifesting as aortic dissection (AD).^4,5,6^ PAU, as well as intramural hematoma (IMH), are managed both conservatively and surgically.^7,8^ Small intimal tears are seen in patients of IMH during surgical exploration.^9^ The symptom of chest pain in a hypertensive patient should alert the possibility of PAU.^10^ Computed tomography angiography (CTA) has made it easier to detect and plan the endovascular approach and its execution for PAU.^11-14^ This article is a review of acute aortic syndrome (AAS) spectrum, mainly PAU and IMH in terms of their natural course, clinical profile, imaging, risk factors, prognosis, outcome, and management strategies. The article search was conducted on the google scholar web search engine using the keyword “penetrating atherosclerotic ulcer of the aorta.” The references from the articles with relevant material on the subject matter were included as subsequent articles.

## Review

### Pathophysiology

PAU is an ulcer invading into aortic media after disrupting internal elastic lamina. The cascade passes through hematoma and pseudoaneurysm, culminating in dissection or rupture.^1^ PAU under the pressure of underlying hematoma dissects through aortic intima and media, leading to pseudoaneurysm formation. The underestimation of aortic involvement in cases of incomplete rupture secondary to thrombus within ulcer crater is also suggested in the literature.^6^ Cystic medial necrosis is suspected to be a potent contributor to AD; however, Larson and Edwards refuted this hypothesis. Their study suggested that PAU rarely leads to intimal tear.^15^ The presence of atherosclerosis has long been an affirmative criterion for PAU, given the lack of histopathological diagnosis. Few studies have therefore named PAU as ‘ulcer-like lesions.^16^ In cases where PAU could be analyzed microscopically, findings indicate degenerated intima with cholesterol deposition, invading the media. Blood products were also seen in media and beneath.^17^ PAU, along with IMH and AD, completes the constellation of AAS. Svensson gave subcategories of AD with classical AD, IMH, and PAU as three of them.^18^ Apart from acquired etiology, AD can be of congenital and familial origin.^19^

IMH was first described as dissection without intimal tear by Krukenberg et al and Yamada et al^20-21^ Vasa vasorum are tiny vessels within smooth muscles that penetrate and provide oxygen to the arterial wall.^2^ The rupture of these vessels secondary to degenerated media is the pivotal step in AD with IMH as a frequent intermediator.^20,22-23^ Sundt et al had contrary beliefs and refuted that vasa vasorum (VV) rupture acts as a starting point of IMH.^14^ Ischaemia of VV due to an increase in arterial pressure and age-related physiological changes has also been proposed to reduce the elasticity of media and contribute to AD.^8-9^ In contrast, the other type has been called a spontaneous variant of IMH as it occurs secondary to blunt trauma, neither associated with atherosclerosis or PAU.^7^ Stanson used the nearly same terminology, with Valente labeling as a variant of AD, having a small intimal defect and thrombosed false lumen without a reentry point.^6,13^ AD is said to have two intimo-medial tears, whereas IMH has a single intimo-medial tear termed entry tear.^2^ The proportion of media in this intimo-medial tear is directly related to the probability of aortic rupture.^24^ Hypertension leads to differential diffusivity of nutrients between aortic layers, contributing to differences in elasticity.^2^ This variable elasticity is also the cited reason for the close segmental association between IMH and PAU.^4^ Typically, aortic diameter progresses by 1-2 mm per year, along with a synchronous increase in wall thickness and stiffness.^25^ The demarcation point between IMH and PAU is the lack of entry point between the media and intima, as seen in the former’s case.^9^ Recently IMH is seen as a small thrombosed tear in the aortic intima precluding their visualization by imaging techniques.^7,26,27^ In a study by Ganaha et al PAU was a causal factor for IMH in just above half of the cases. The association of PAU was strongly correlated with type B IMH. They also shared the same distinction among cases of isolated IMH and those associated with PAU.^7^ The study by Cho et al and Batt et al supported this observation.^5,16^ However, Stanson et al had not shared the same views.^6^

Loss of elasticity is the crucial element in the senile aorta, with smooth muscles, elastin fibers, and lamellae all playing their part. With aging, there is an increase in thickness, collagen content, and macrophage count within aortic layers. At the molecular level, there is altered synthesis and expression of tropoelastins and metalloproteins (MMP). MMP-12 is the culprit in inciting atherosclerotic plaque instability, whereas MMP-2,9,12 are involved in aneurysm formation.^28-29^ Plasminogen activator inhibitor-1 (PAU-1) and messenger ribonucleic acid (mRNA) are exhibited across all phases, plasminogen activators (PAs), urokinase-type plasminogen activator (u-PA) and tissue-type plasminogen activator (t-PA) and MMP-9, are expressed in the subacute phase, whereas chronic phase is dominated by PAI-1 mRNA with negligible detection of u-PA and t-PA.^30^ Inflammation inciting expression of MMP leads to medial degeneration with apoptosis adding to the cascade. Ganaha et al also classified patients as having progressive or stable disease course depending upon the behavior of IMH on follow-up imaging.^7^ Indicators favoring IMH resolution were younger age, aortic diameter around 4 cm, IMH thickness less than 1cm, enhancement of false lumen, and usage of beta-blockers in the postoperative period.^31-34^ Apart from atherosclerosis, traumatic and iatrogenic causes also incite IMH formation.^35^

PAU leading to AD is resisted by many, including the viewpoint of critical intimal fibrosis containing ulceration and limiting the extent of IMH.^1^ The same fibrotic changes were held responsible for stable aortic lumen obviating end-organ ischaemic fatality.^9,12^ The study by Coady et al projected a higher rate of rupture in the case of PAU, to the tune of 42%, slightly higher than in cases of IMH and AD individually. They postulated a higher probability of PAU rupture with aortic diameters of more than 6.5 cm and ulcer depth of more than 1cm, a statement against the consensus.^17^ The higher rate of rupture was also correlated with the disruption of the aortic aneurysm.^36^ The study by Lee et al supported the same view citing cases of PAU localized to the arch showing no signs of rupture. The reason quoted is the dimension of both aorta and PAU well below the defined limits.^1^ However, the same does not hold valid for IMH, the prognosis of which is intricately related to AD that it is classification in similar lines of AD.^7^ Cases of AD complicating PAU tend to be localized, have a higher frequency of type B dissection, a retrograde morphology, and a true lumen of comparative size as the false lumen.^24^ In a rare scenario, PAU may lead to extensive dissection, a realistic possibility in cases centered over the arch.^37^ The presence of IMH significantly elevates stress on aortic media, considerably increasing the likelihood of frank dissection or rupture.^25^ In another scenario, IMH may involve and cause rupture of the origin of adjacent intercostal, lumbar, or bronchial arteries leading to the formation of intramural blood pool (IBP). They represent intramural hematoma communicating with side branch entry point via a narrow orifice.^9,38^ IMH may be localized or extensive, defined by the involvement of two or more aortic segments.^5^ As part of natural history, IMH may show dimorphic early and late progression. In one study, rapid progression contributed three-quarters of cases, with the most commonly encountered finding being contained rupture. Paradoxically, the mortality was higher in the group with the slow progression of the disease.^39^ The latter group may lead to either double-barreled or more commonly encountered thrombosed morphology of aortic dissection.^10,40^

The predisposing atherosclerotic changes hinder double-barrel morphology due to loss of vascular pliability. Roberts WC was also of the opinion that atherosclerotic changes like atrophy of aortic media and associated fibrotic changes are actually deterrents in the formation of AD.^41^ Focal intimal disruptions (FID) is a closely related condition seen during the evolution of the atherosclerotic process.^9^ With PAU being recognized as a solitary lesion, seen on the initial study, devoid of any intimal flap or IMH, FID contrasts in being known anywhere between 2 weeks to 6 months, shows the variable extension of IMH and intimal flap in later stages.^4^ Limited intimal tears (LITs) were the newest addition to the spectrum of AAS discovered by Murray and Edwards in 1973 in an autopsy series. It was reemphasized in 1999 by Svensson et al during repair of ascending aortic lesions which was later classified as class III intimal tear. By far apart from these two studies, it was concluded that this entity was either missed initially or misclassified.^42^

Spontaneous AD, starts at the point of highest shear stress along the counter facing walls of the aorta inciting intimal tear and culminating into the frank dissection. Pressure atrophy of media due to atherosclerosis and localized bulge in aortic contour facilitate the same.^40^ A study on computed models of the abdominal aneurysm by Vorp et al showed that stress on aneurysmal wall increases in a non-linear manner dependent on both maximum diameter and shape of the aneurysm. The point of maximal pressure changes from the midpoint of the posterior to the anterior surface as the aneurysm enlarges. This dynamic phenomenon envisages the fact that the diameter of an aneurysm should not be the sole criteria guiding intervention.^43^ A case highlighted the same mechanism where PAU was seen at the point of maximum ectasia, a similar mechanism in spite of no significant underlying atherosclerotic changes.^44^

### Clinical features

Hypertension, hyperlipidemia, and coronary artery disease (CAD) are the strongest clinical and laboratory attributes of PAU.^1^ The patients are invariably active or past smokers.^24,40^ The same factors hold for IMH. The clinical presentation of PAU is similar to AD except for valvular, cardiac rhythm abnormalities, and ischemic tendencies being seen frequently in the latter.^2,45^ Other atypical findings in the case of PAU are pulse abnormality, signs of a stroke, vascular insufficiency, and end-organ infarction.^6^ Pain in the chest, especially radiating to the back, found to be one of the strongest predictors of PAU rupture.^4,6,12,36^ The radiation of pain may suggest the site of the lesion, anterior chest pain, indicating ascending and pain in back for descending aortic lesions.^36^ Intermittent chest pain radiating to the shoulder and back can be another manifestation of the disease, with recurring pain indicating impending rupture.^46,47^ The presence of pleural effusion and a long segment of IMH involvement was frequently seen in symptomatic cases, whereas microembolisation events alerted towards the same in asymptomatic cases.^5^ Even in the absence of pain, approximately one-third of patients progressed to aneurysm formation over a seven-year follow-up.^12^ In many patients, PAU is encountered as an incidental finding devoid of any clinical manifestation whatsoever while investigated for an unrelated condition. Table 1 summarizes the clinical profile in terms of age distribution and gender of patients, frequency of substance abuse, aortic abnormalities like dissection or rupture, and other associated vascular abnormalities.

**Table 1:** General profile

**Study**	**Year**	**Number of patients**	**Age Range (years)**	**Gender (M:F)**	**Associated comorbidities**	**Substance abuse (%)**	**Symptomatic (%)**	**Associated aortic abnormality**	**Aortic dissection/ rupture (%)**	**Others**
Stanson et al^6^	1986	16	74+10	8:8	HTN, CAD, Hyperlipidemia	11 (68) –Smoking	13 (81)	3 – TAA	13 (81)	4 – postoperative paraplegia 3 – prior CVA 1 – prior vascular surgery
Kazerooni et al^39^	1992	16	73+15	7:9	HTN, CAD, PVD, CVA	-----	13 (81)	6 – Aneurysm	1 (6)	1 – perioperative CVA
Harris et al^12^	1994	18	74+12	10:8	HTN, CAD, DM, CKD	-----	4 (22)	12 - Aneurysm	0 (0)	2 – distal foot ischemia 1 – lower limb DVT
Coady et al^17^	1998	19	74+11	9:10	HTN, CAD, COPD, CKD, DM	-----	16 (84)	8 – AAA	8 (44)	-----
Vilacosta et al^24^	1998	12	65+10	12:0	HTN, CAD, Hyperlipidemia	10 (83) – Smoking	12 (100)	2 – TAA	6 (50)	2 – syncope 1 – MI
Hayashi et al^10^	2000	12	70+5	11:1	-----	-----	6 (50)	2 – TAA 4 – AAA	2 (16)	1 – died of unrelated disease
Quint et al^58^	2001	38	75+20	20:18	-----	-----	22 (58)	-----	2 (5)	1 – AKD 1 – sepsis/CLD/AKD
Ganaha et al^7^	2002	65	69+10	34:31	HTN	-----	63	10 – TAA 5 – AAA 8 – TAA+ AAA	663 (91)	1 – hemoptysis 1 – paraplegia
Tittle et al^59^	2002	45	71+17	18:27	-----	-----	45 (100)	-----	34 (75)	2 – unspecific non-cardiovascular cause 2 – MI 1 – CVA 1 – COPD
Cho et al^5^	2004	105	72+9	73:32	HTN, COPD, CKD, CAD	81 (77) – Smoking	79 (75)	64 – AAA	9 (9)	1 – mycotic involvement 1 – acute paraplegia
Batt et al^16^	2004	8	72+6	7:1	HTN, COPD, CAD, CVA, PVD, DM	8 (100) – Smoking	6 (75)	1 – TAA 2 – AAA 1 – CIAA 2 – PAA	3 (37)	4 – SAP 2 – Renal artery stenosis 1 – acute lower limb ischemia 1 – SMA thrombosis 1 – IMA implantation
Piffaretti et al^82^	2006	13	73+7	12:1	HTN, DM, COPD, CAD, CKD, Hyperlipidaemia	-----	10 (76)	-----	-----	5 – thrombus in AA 5 –iliac artery stenosis 4 – extensive aortic calcifications 2 –internal carotid artery stenosis
Brinster et al^73^	2006	21	73+12	7:14	HTN, DM, COPD, CAD, CKD, CHF, Hyperlipidaemia	16 (76) – Smoking	16 (76)	-----	4 (19)	4 – previous CVA 2 – pulmonary embolism 2 – previous DVT
Piffaretti et al^83^	2007	11	68+13	9:2	HTN, COPD, CKD, IHD	-----	6 (54)	3 – concomitant AAA 2 – repaired AAA	4 (36)	3 – acute renal failure 1 – transient ischaemic attack
Kuehl et al^62^	2008	33	66+20	28:5	HTN, CAD, DM	-----	-----	5 – TAA	14 (42)	-----
Patel et al^57^	2010	37	72+10	16:21	HTN, COPD, CAD, CVA, PVD, DM	20 (54) –Smoking	36 (97)	1 – TAA	15 (40)	2 – perioperative stroke 2 – temporary paraplegia 1 – transient renal failure requiring dialysis
Nathan et al^11^	2012	315	70+10	234:81	HTN, COPD, DM, CAD, Hyperlipidaemia, CKD	-----	69 (21)	288 – TAA/ AAA	16 (0.05)	-----
Patel et al^86^	2012	95	70+9	44:51	HTN, CAD, PVD, DM	Smoking – 54 (56)	-----	24 – repaired AAA	34 (35)	8 – infected aortic pathology
Salim et al^76^	2019	43	66+25	26:17	HTN, COPD, DM, CKD, IHD	Smoking – 23 (53)	16 (37)	-----	-----	11 – aortic surgery for non-PAU disease

Abbreviations: HTN, hypertension; CAD, coronary artery disease; TAA, thoracic aortic aneurysm; CVA, cerebrovascular accident; PVD, peripheral vascular disease; DM, diabetes mellitus; CKD, chronic kidney disease; SAP, subadventitial pseudoaneurysm; SMA, superior mesenteric artery; DVT, deep vein thrombosis; COPD, chronic obstructive pulmonary disease; AAA, abdominal aortic aneurysm; MI, myocardial infarction; AKD, acute kidney disease; CLD, chronic liver disease; IHD, ischaemic heart disease; CIAA, common iliac artery aneurysm; PAA, popliteal artery aneurysm; IMA, inferior mesenteric artery; CHF, congestive heart failure

### Imaging findings

A chest radiograph is the first modality invariably undertaken in a case of chest pain. Patients with IMH and PAU have unremarkable chest radiographs as compared to findings of mediastinal widening with or without pericardial effusion in cases of AD.^4,48^ Other radiographic indicators for the presence of AD were haziness in the left lung apex or left hemithorax and left-sided tracheal deviation.^39,49^ Prominent bronchovascular markings are another manifestation representing mediastinal hematoma extending into the pulmonary interstitium.^50^

CTA is the imaging modality of choice for evaluation of AAS being faster, less invasive, requiring less technical expertise, and ability to reproduce images in any plane with excellent resolution. CTA should be performed after clinical and laboratory evaluation, including cardiac enzymes and D-dimer assay, chest radiograph, and electrocardiogram.^24^ Guidelines regarding indications of CTA (intermediate and high-risk categories), clinical evaluation, and technique to perform CTA in cases of suspected AAS are laid down in 2016.^51^ Their main emphasis was to acquire motion artifacts free images, especially of the aortic root with ECG gating. End-systolic versus end-diastolic acquisition depends on the patient’s heart rate and the number of the detector array. Recommendations included coverage limited to thoracic aorta, the addition of a non-contrast sequence to detect any associated hematoma, and targeting 250 HU or more attenuation value in the arterial phase.

The intimal flap of dissection and associated intramural hematoma is not evident on aortography as seen on CTA. Instead, indirect signs like medial displacement of intimal calcification can be a clue for the same.^6,14^ On CTA, the distinction between true and false lumen can be made reasonably; however, it can be tough in cases where the entire aorta is not included in the scan. The interface between intensely enhancing true and crescentic false lumen can give a beak-like morphology. Acute cases may show outer wall calcification and convex flap morphology towards the true lumen. False lumen shows characteristic cobwebs in the acute stage with a relatively larger cross-sectional area in the chronic phase.^52,53^ Pulsation artifacts can be confused with intimal flap; specific location and visualization in one or two sections can eliminate this confusion.^54^ With advancements in imaging, diagnosis of PAU and IMH can be made with better confidence, transoesophageal echocardiography (TEE) leading from the front.^53,55^

### Penetrating atherosclerotic ulcer

The distinction between PAU and sinister AD is vital with the site of the lesion, presence of intramural hematoma, and intimal flap providing a good demarcation improved by dynamic contrast-enhanced imaging.^1,39^

**Figure 1:**
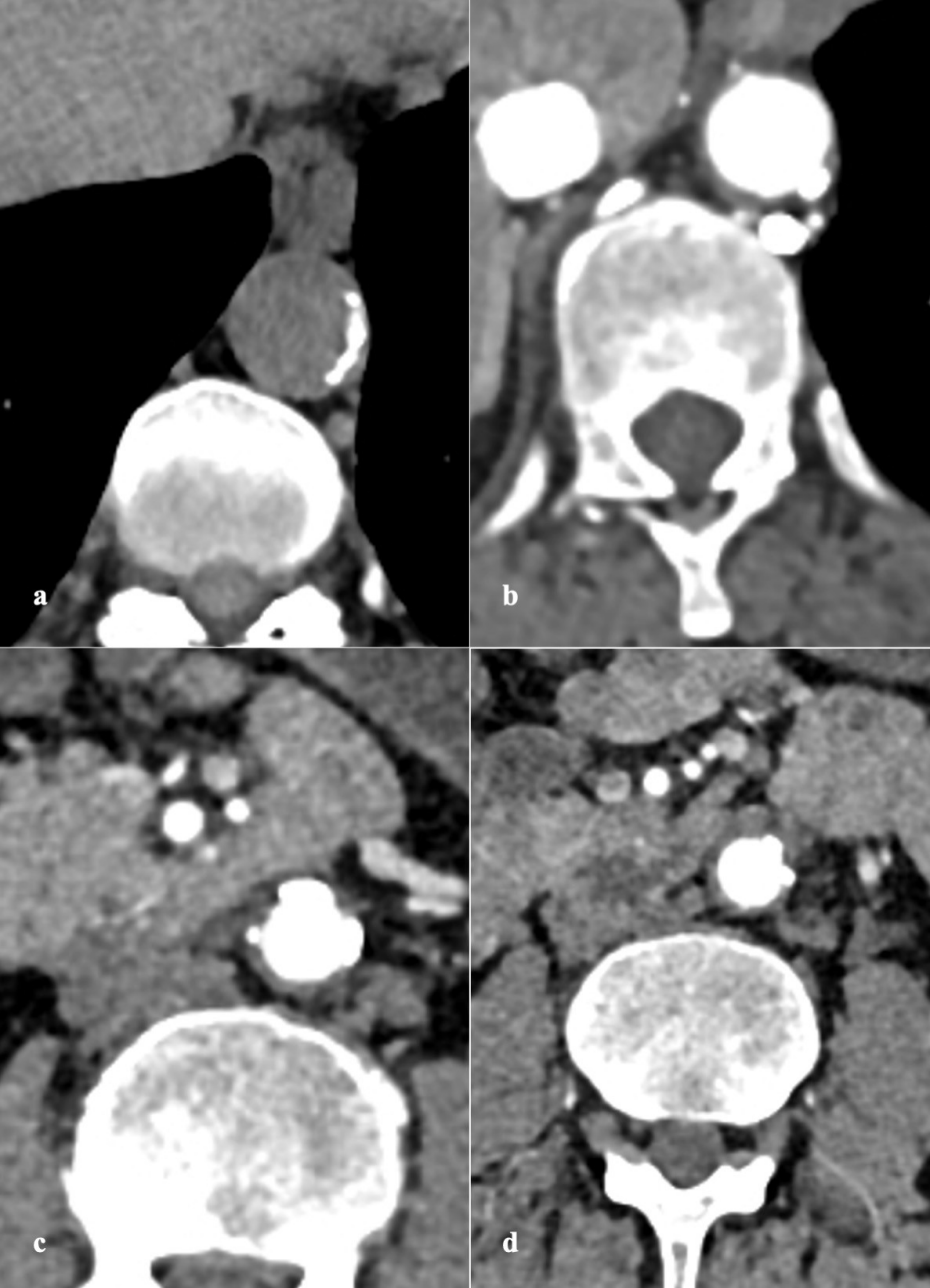
Axial plain (a) CT image shows calcified atherosclerotic wall calcification. Axial arterial phase (b,c,d) CT images reveal multiple contrast-filled outpouchings arising along all walls of the aorta, suggesting penetrating atherosclerotic ulcer

**Figure 2:**
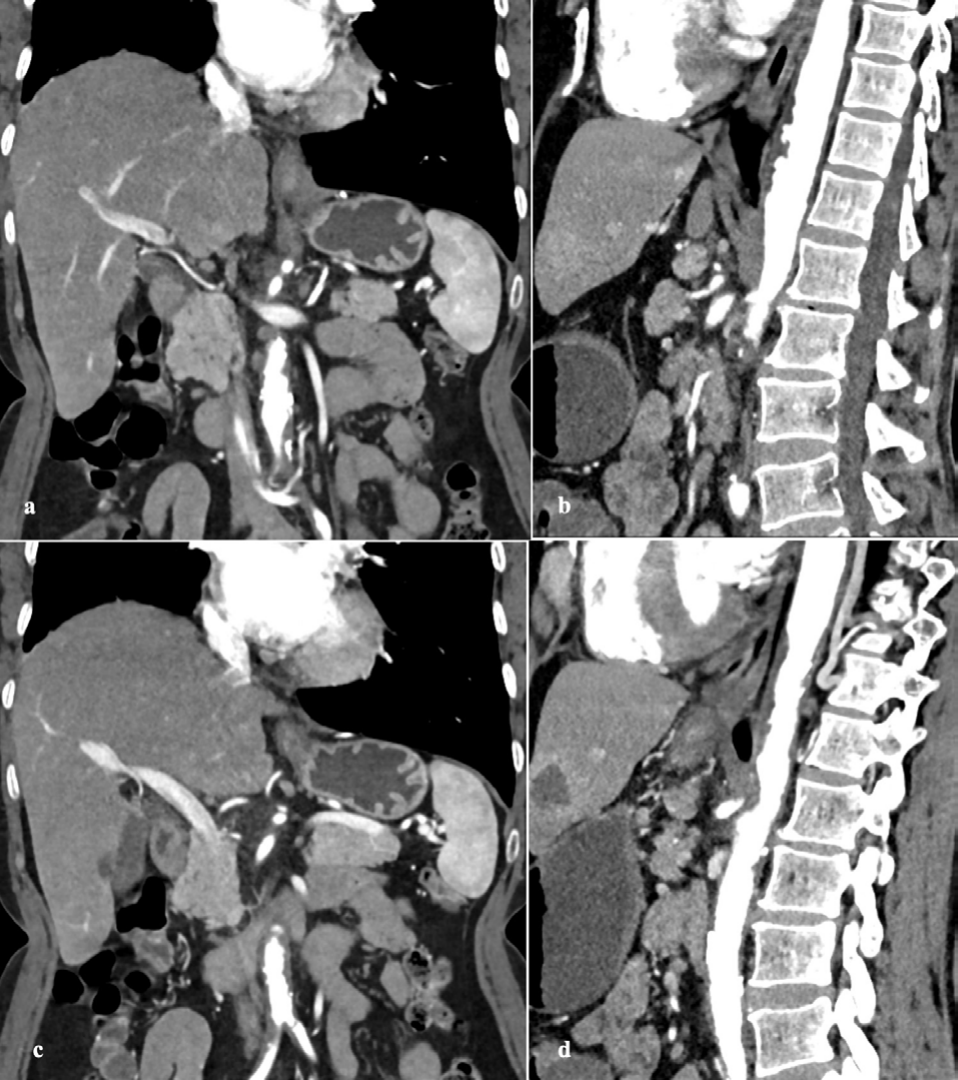
Coronal (a,b) and Sagittal (c,d) multiplanar reconstructed arterial phase CT images depict the true extent of penetrating atherosclerotic ulcers

**Figure 3:**
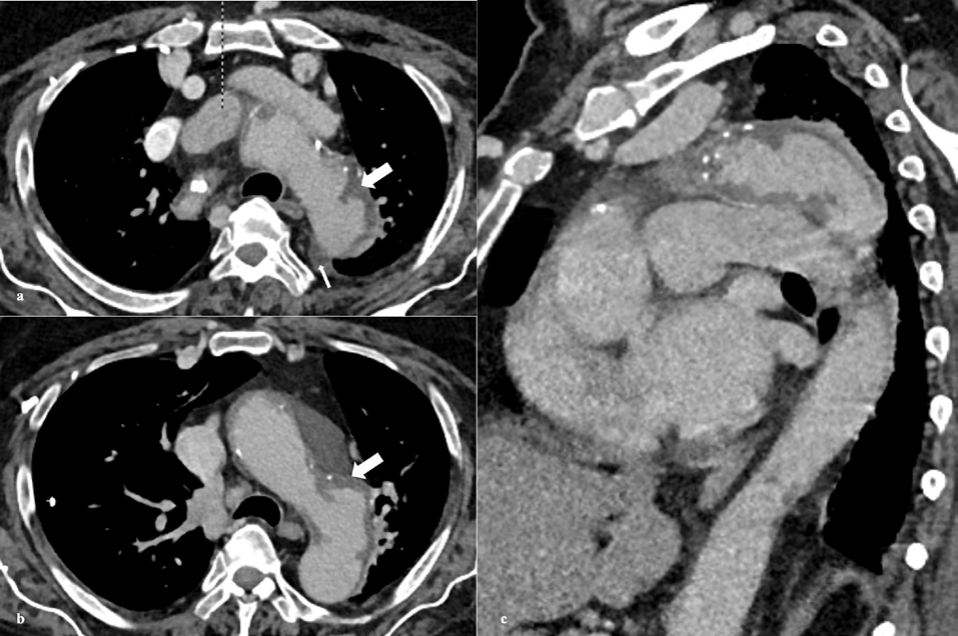
Axial (a) and Sagittal (b,c) contrast-enhanced CT images reveal penetrating atherosclerotic ulcers involving aortic arch with associated intramural hematoma (thick arrow) and small effusion (thin arrow)

PAU is seen on CTA as contrast filled outpouching (Figures 1, 2) or crater-like morphology on aortography and TEE.^4,6,56^ PAU ranges in size from few millimeters to 2.5 cm, depth up to 3 cm, are often multiple.^36^ There is invariable surrounding IMH and medially displaced calcified intima (Figure 3).^8,10^ A study by Mayo clinic confirmed this association to the tune of 80 percent.^5^ Hyperdensity in PAU on non-contrast study denotes intimal hematoma, an indicator of acute and potentially unstable state warranting prompt intervention.^13^ The adjacent aortic segment is invariably thickened with some degree of enhancement.^1,39^ An interesting differential of PAU involving abdominal aorta is inflammatory aneurysm characterized by marked wall thickening and associated fibrosis forming surrounding adhesions.^56^

The conventional angiographic and CTA appearance of PAU is similar to a gastric or duodenal ulcer seen in barium studies.^6,13,17^ PAU usually does not extend beyond the aortic contour, latter being suspicious for rupture, associated hematoma or Subintimal pseudoaneurysm formation.^16^ Magnetic resonance imaging (MRI) appearance is akin to the area of flow void showing flow-related enhancement on time-of-flight sequence. Increasing the TE would further enhance the detection of sluggish flow in ulcer crater.^12^ Nonfat sequences were technically less demanding and more accurate than fat saturation sequences and even CTA.

PAU needs to be differentiated from both IBP and ULP (ulcer-like projection), with the former having a wider diameter and intimal atherosclerotic changes.^34^ ULP is commonly encountered on follow up imaging in patients with the normal aorta.^2^ False-positive cases were attributed to focal wall calcification mimicking flow void, whereas sluggish blood flow was false-negative diagnosis was attributed to in the same study. The same was realized when demarcating between PAU and entry tear associated with IMH mimicking PAU. Hence, many authors have included both terms under the umbrella term of PAU.^12,57^ Similarly, ULP can be differentiated from IBP, with the former having a full luminal diameter of 3mm or more.^38^ The disruption of the internal elastic lamina is the key histological finding in cases of PAU, which can be seldom demonstrated.^24^

PAU is most commonly seen in descending thoracic aorta followed by abdominal aorta and arch in decreasing order of frequency. The symptomatic disease was also more common in the thoracic segment of the aorta.^10-12^ Detection of PAU on imaging is difficult in cases localized to ascending aorta, the diameter of less than 10mm, and those with no demonstrable contrast leak.^48^ The disease progression can be defined on the basis of an increase in the dimension of PAU, aorta, or both. Nathan et al found the progressive nature of PAU in around 25% of cases on follow-up. The progressive nature of the disease was not affected by PAU diameter, associated IMH, aortic diameter, or aneurysm formation, if any.^11^ Noticeably they deduced their study conclusions taking CTA as a standalone imaging modality. The same views were shared by Harris et al regarding the lack of imaging factors predictors of PAU progression.^12^

The rupture of PAU was seen in around 13%-14% of cases, as mentioned in studies by Nathan and quint.^11,58^ The highest number of PAU rupture encountered in literature was by Tittle et al quoting at 38%. This unusually higher percentage was attributed to all cases being symptomatic and half of the cases localized to the ascending aorta.^59^ Isolated PAU was more commonly seen in males, whereas PAU with IMH was more prevalent in females.^11^ In a study by Tittle et al there was a female predominance for both PAU and IMH.^59^ FDG-PET (fluorodeoxyglucose-positron emission tomography) has also been used to evaluate the inflammatory component of PAU. It has been shown to be helpful in predicting vascular events in the brain and aorta.^60-62^ Table 2 summarizes salient findings in cases of PAU in terms of its distribution among different aortic segments, multiplicity, and a maximum diameter of the native aorta.

**Table 2:** Imaging findings

**Study**	**Year**	**Number of patients**	**Maximum diameter of the aorta (mm)**	**PAU**				**IMH**			**Pleural effusion (%)**
				**Presence (%)**	**Distribution** **(As:Ar:D)**	**Multiple (%)**	**Presence (%)**	**Stanford type (A:B)**	**Maximum thickness (mm)**	**Localised (%)**	
Stanson et al^6^	1986	16	-----	16 (100)	1:1:14	5 (31)	15 (93)	2:14	20	8 (50)	1 (6)
Kazerooni et al^39^	1992	16	-----	16 (100)	0:1:15	1 (6)	16 (100)	-----	-----	-----	7 (43)
Harris et al^12^	1994	18	-----	18 (100)	0:1:18	11 (61)	-----	-----	-----	-----	3 (16)
Coady et al^17^	1998	212	62+15	19 (9)	2:2:17	-----	17 (8)	5:12	-----	-----	-----
Vilacosta et al^24^	1998	12	-----	12 (100)	1:2:3	1 (8)	1 (8)	-----	-----	-----	-----
Hayashi et al^10^	2000	12	-----	12 (100)	0:0:12	12 (100)	-----	-----	-----	-----	-----
Quint et al^58^	2001	38	51	38 (100)	1:14:35	1 (2.6)	22 (58)	-----	-----	-----	5 (13)
Ganaha et al^7^	2002	65	47.4+8.3	34 (9)	-----	-----	65 (100)	28:37	-----	-----	20 (30)
Tittle et al^59^	2002	45	59	26 (57)	12:0:14	-----	19 (42)	11:8	-----	-----	-----
Cho et al^5^	2004	105	43.4+7.9	105 (100)	0:9:99	11 (10)	85 (81)	22:83	9.7+4.0	49 (58)	32 (30)
Batt et al^16^	2004	8	-----	8 (100)	8 – AA	-----	-----	-----	-----	-----	-----
Piffaretti et al82	2006	13	-----	13 (100)	13 – AA	-----	-----	-----	-----	-----	-----
Brinster et al^73^	2006	21	-----	21 (100)	-----	-----	-----	-----	-----	-----	-----
Piffaretti et al^83^	2007	11	60	9 (81)	0:5:4	-----	-----	-----	-----	-----	2 (18)
Kuehl et al^62^	2008	33	-----	8 (24)	-----	-----	6 (18)	-----	-----	-----	-----
Patel et al^57^	2010	37	50+14	37 (100)	-----	-----	19 (51)	-----	-----	-----	-----
Nathan et al^11^	2012	315		315 (100)	0:28:240	73 (23)	56 (17)	-----	-----	-----	-----
Patel et al^86^	2012	95	58+15	95 (100)	-----	-----	41 (43)	-----	-----	-----	-----
Salim et al^76^	2019	43	35.6+8.6	43 (100)	0:11:32	9 (20)	-----	-----	-----	-----	-----

Abbreviations: PAU, penetrating atherosclerotic ulcer; IMH, intramural hematoma; Ar, arch; As, ascending;

### Intramural hematoma

IMH is seen on CT as a crescentic hyperdense non-enhancing area of high attenuation, showing a linear and tangential orientation. In the majority, there is no luminal compromise or displacement of intimal calcification in contrast to classical AD.^13^ Contrast enhancement when present can attribute to the presence of IBP or ULP, with the former having narrow and the latter having a broad neck.^38^ The appropriate slice thickness is 5mm with multiplanar reconstruction using a small window width.^2^ Whenever IMH is encountered during an imaging study, the points to be reported include its thickness, any focal enhancement or ulceration, periaortic hematoma, the diameter of the aorta, and the presence of pleural or pericardial effusion.^2^ Aortic wall thickness more than 5mm implies the existence of IMH in an appropriate clinical context; however, the same can be up to 10mm.^25^ When IMH exceeds 10mm in thickness, there is an association with the presence of IBP in the descending thoracic aorta.^38^ Irregularity of outer contour of the aorta, mediastinal hemorrhage, and increasing distance between aorta and esophagus also increase the probability.^42^

MRI is superior to CT in the detection of IMH, precluding the use of contrast material due to high contrast resolution.^10^ MRI imaging appearance is of a thickened aortic wall showing variable signal intensity depending on the stage of blood products. Cine images can also help in differentiating the slow flow of AD from absent flow in IMH.^8,63^ The corresponding TEE appearance ranges from echogenic to echolucent, devoid of any intimal flap and 7mm or more in thickness.^35,64^ TEE provides a better assessment of intima and visualization of small tears; MRI, on the other hand, has the advantage to display associated mediastinal hematoma.^2^ IMH is usually not diagnosed on aortography as the luminal surface of the aorta is intact.^40^ As compared to AD, IMH is seen in the older age group; however, the maximum thickness of IMH can predict the occurrence of AD.^65^

Halapas et al reported a case of Takayasu’s arteritis mimicking IMH on both TEE and per operative epiaortic ultrasound.^66^ On resection, marked aortic wall thickening was appreciated with the presence of necrobiotic foci and reactive inflammation on histopathological examination. Another pitfall is the thrombosed false lumen of dissection, which can be confirmed during surgery only; however, spiral morphology along the length of the aorta being a favoring point.^13^ IMH in the vicinity of the left coronary ostium can be mistaken for incomplete dissection with associated subadventitial hematoma.^2^ In sharp contrast, true dissection shows frequent involvement of the right coronary ostium.^47^ Aortic intimal sarcoma has a lobulated contour and may extend beyond the aortic outline, differentiating it from IMH. Apart from contrast related and motion artifacts, several anatomical structures on axial images can mimic AD. Origins of the brachiocephalic trunk, left brachiocephalic vein, left superior intercostal, left inferior pulmonary veins along with left lung collapse consolidation mimicking Stanford B type AD. PAU is the most crucial pathological impersonator of type B dissection. Right atrial appendage and superior pericardial recess can mimic type A dissection. In children, thymic tissue can be mistaken for the false lumen of dissection.^54^ Table 2 summarizes salient findings in IMH cases of PAU in regard to its maximum thickness, localized status, and aortic distribution in terms of Stanford classification.

### Prognosis and outcome

PAU diameter of 20 mm and depth of 10mm when taken as cutoff, predicted disease progression, suggesting early surgical intervention reasonably.^7^ PAU has the worst prognosis in cases of rupture, leading to hemomediastinum and or hemopericardium.^6^ Likewise, rupture at initial presentation and maximum aortic diameter predicted the failure of medical treatment.^14^

The occurrence of PAU with IMH generally leads to a progressive disease course with a higher likelihood of catastrophic consequences like aortic rupture and dissection.^7,33^ These patients usually belong to an older age group and show involvement of the proximal thoracic aorta. The predictors of disease progression were pain despite expectant treatment, increase in pleural effusion, and disease confined to the proximal thoracic aorta. A higher subset of symptomatic patients explained the same.^7^ The presence of pain, hemodynamic instability, suboptimal response to medical treatment, IMH thickness 11 mm or more, periaortic hematoma, and associated PAU beyond a particular dimension are all predictors for rupture.^2^ In the context of type A IMH aortic wall thickness of 12mm at two weeks of admission predicted rupture with a 100% negative predictive value.^67^ Interestingly, Cho et al and Batt et al were not able to predict the patient outcome based on pain or the presence of IMH.^5,16^ Quint et al found the absence of pleural effusion to be the only CT predictor of disease stability.^58^ Pleural effusion arises either through micro-perforation or inflammatory reaction of the aortic wall. Vorp et al added that apart from diameter, shape and asymmetry of aneurysm are equally strong predictors for rupture.^43^ Mean aortic diameter more than 40mm is considered a risk factor for IMH progression, with that over 50mm correlating with mortality. Table 2 summarizes the prevalence of pleural effusion in cases of AAS, especially PAU and IMH.

The aortic diameter predicting rupture can also be stratified depending upon the site of IMH, with higher values for Stanford A than B type IMH concluding poor prognosis and higher mortality in the former group.^2,14^ Interestingly, type A IMH was not a predictor of mortality as these cases underwent early surgical repair and were detected before 40 years of age.^19,47^ Advance age at first diagnosis confers a better prognosis owing to atherosclerotic changes giving a protective effect on IMH extension.^35^

LITs are the entity of concern by being challenging to diagnose and invariably first seen on surgery, having the potential to convert into the frank lethal dissection.^42^ IBP is a relatively benign entity, those having a significant diameter and communicating with intercostal or lumbar artery leading to incomplete resorption.^2,33,38,55^ The prognostic significance of FID is not clear.

Approximately one-third of cases of AAS showed significant uptake on FDG-PET, denoting vessel wall inflammation, which signifies disease progression. This correlation gets further stronger in analyzing FDG-PET with D-dimer assay.^62^

The long-term prognosis of patients depends upon the control of blood pressure and keeping other risk factors under control. The same was favored in a study predicting favorable outcomes in patients of IMH treated with beta antagonists. The highest risk of emboli is present in cases with ulcerated or mobile plaques.

## Complications

Two cases of ascending aorta PAU complicated by rupturing into the pericardial cavity and leading to hemopericardium.^64,68^ Cardiac tamponade, aortic regurgitation, and left-sided haemothorax are less common sequelae of PAU.^3,49,64,69^ Kazerooni et al described a case of contained extrapleural hemorrhage secondary to leaking penetrating ulcer in the thoracic aorta.^39^ PAU rarely forms fistulous communication eroding into the esophagus, stomach, and duodenum, manifesting as catastrophic gastrointestinal bleeding and very high mortality.^70-72^ This type of fistulous communication also anticipates high rates of stent-graft infection.PAU localized to the abdominal aorta show less likely to rupture, paradoxically have a higher tendency to aneurysm formation and lower extremity embolism than their thoracic counterpart.^16^

Vascular complications are by far most common in AD among the subsets of AAS. Distal embolization leads to vascular insufficiency and, if not resolved, end-organ infarction.^13,36^ AD of descending aorta can present as paraplegia, with remedial measure proposed for the same being intercostal artery revascularization in one of the studies.^6^ In a rare instance, AD complicating haemopericardium showed no signs of tamponade, spontaneously recovered, and survived for the next three months. This so-called healing leads to the communication of aneurysm with native lumen at two points, giving the term - double aorta.^3^

## Management

The management of PAU is guided by the symptoms, involved aortic segment, imaging indicators of progression, and associated entities. As far as medical management is concerned, it is targeted to reduce mechanical stress on the aorta using beta-blockers. Hemodynamic instability, persistent or increasing pain despite treatment, larger aortic diameter >55-60 mm or aneurysm formation, a continual increase in diameter, contained or frank rupture, hemopericardium, or large hemothorax are established criteria to undertake surgical approach irrespective of the involved aortic segment.^4,6,7,10,39,56-60,64-65,73-75^ In cases of PAU localized to ascending aorta and/or arch urgent surgical management whether open surgical or thoracic endovascular aortic repair (TEVAR) is warranted. There is no uniform consensus as to when to choose between expectant and surgical management in cases of PAU localized to descending aorta. PAU complicated by subadventitial aneurysm, presence of acute FID, enlarging IBPs, bronchial compression or aortobronchial fistula, and signs of distal embolization require urgent surgical management either open surgery or TEVAR. ^2,4-6,8-9,16,18,23,33,55^ TEVAR is preferable in patients showing signs and symptoms of abdominal or limb ischemia, those with associated infectious aortitis especially mycotic and anatomical abnormality like coarctation.^18,76^ Cho et al suggested medical management for PAU and also expected resolution of IMH with time.^5^ This approach was supported by other studies in regard to asymptomatic cases and those involving descending aorta.^12,17,36,46,76^ When the IMH is seen in concurrence with PAU, urgent surgical management is an absolute indication in cases of Type A IMH. For Type B IMH the indications remain more or less the same as those for PAU.^14,77^ There is no survival advantage of TEVAR when compared with open surgical treatment with two years as a comparative endpoint. This comparable prognosis of TEVAR with open surgical management can be partly explained by the fact that the latter also addresses the treatment of comorbidities, for instance, CAD.^18^ However, the majority of studies stressed follow up imaging acknowledging disease progression, leading to late complications and surgical management.^6,11-12^

### Open versus endovascular repair

The choice between open and endovascular approach depends on the patient’s age, associated comorbidities, technical factors including lesion morphology and anatomical variations, available prosthesis, and centers expertise.

Earlier, AD alone used to be treated by stent-graft placement at the site of the tear, with cases having associated PAU or type A IMH, the same lesion when included showed a favorable response.^6,78^ In cases of TEVAR, accurate deployment of the stent can be challenging due to pulsatility and pressure of flowing blood.^79^ To overcome it maintaining systolic blood pressure in the 50-65mm Hg range using vasodilators and or beta-blockers was proposed.^80^ Technical factors are also limiting, with a complicated delivery system, graft size mismatch, complications like stent migration and penetration, perigraft leak, and transient cord ischemia all contributing.^77,79,81^ Hardware related technical factors considered in one study were including up to 50% wall aortic wall thickness and oversizing stent diameter by up to 10%. This is done considering initial reduction followed by an increase in aortic diameter as IMH undergoes resolution.^57^ An open repair can be performed by either resection anastomosis or segmental aortic replacement with a graft, preferred in cases of the dilated ascending aorta.^44^ The open repair has overall higher mortality with endovascular technique approached 100% success with nil mortality.^42,73,79,82-85^ TEVAR has a noticeable advantage in cases undergoing early repair; however, patient’s age and acuity of presentation predict mortality in cases of late repair.^86^ Semba et al successfully performed stenting in 11 cases of acute rupture within the first week of presentation.^80^ The concurrence of these studies was reflected in increasing acceptance of TEVAR is the preferred method over open repair.^87,88^

TEVAR is preferred in asymptomatic patients, those with large size of associated PAU, those with a progressive aneurysm, and a sizeable pleural effusion.^4,7,45^ Aortic tissue in patients with PAU is usually friable, making open graft repairs difficult.^76^ Relatively normal aortic morphology on either side of the ulcer facilitates acquiring landing zones for better graft stability.^42^ The minimum length of this landing zone required is 1.5-2 cm at either end.^81^ In cases of the long segment of aortic involvement, the use of multiple stents is inevitable to cover the entire abnormal segment and prevent reentry of blood.^81^ In such a scenario, especially with concurrent IMH, balloon dilatation of the stent-graft should be limited so as to prevent graft eroding into the already friable aortic wall.^57^ TEVAR has excellent short and midterm outcome in cases of PAU confined to descending thoracic aorta.^68,73,88^ Apart from the promising outcome,higher incidence of complications like perioperative stroke, permanent cord ischemia, respiratory compromise, and renal dysfunction in open repair are encouraging for TEVAR.^84,86^

In cases of the abdominal aorta, the treatment largely depends on the clinical presentation of the patient as well as the presence of any comorbidities precluding open surgical treatment. TEVAR is the first option in cases presenting as rupture as well as those with high-risk status due to comorbidities.^16,71^ The presence of IMH seems to delay the effectiveness of the treatment, although the risk of endoleak or stent migration is negligible.^56^ Stent grafts provide an advantage in terms of obviating embolic events by preventing the migration of debris or fragments. TEVAR also has a particular advantage in cases of paravisceral PAU, with the placement of branching grafts.^76^ Stent-graft is at a certain disadvantage in cases of PAU complicated by the formation of aorto-enteric fistula, leading to graft infection and potential recurrence of fistulous communication. In such cases, endovascular procedure plays the role of an intermediate step bridging acute presentation with the definite open surgical treatment performed at a later date.^71^ The open surgical procedure is also preferred in the younger age group, which has a relatively lower risk making the former comparable with TEVAR.^56^

The follow-up of symptomatic patients should be done at 1,3,6 and 12 months followed by every year if the disease remains stable. For asymptomatic cases, the follow-up can be done six monthly for the first three years, followed by annual surveillance.^45^ The same should be done similarly for cases having associated FID or aortic dilatation.^4^ The follow-up imaging is even more important in cases of concurrent PAU and IMH, even if it a technically successful procedure. The realistic risk of false lumen formation propagating to dissection are points of concern.^84^

Overall, the early surgical treatment is apt for patients showing the presence of IMH and or PAU in ascending aorta or arch, with TEVAR risking myocardial ischemia and valvular damage as concerns.^7,49^ Re-intervention rate was higher in symptomatic patients, those with dissecting than the non-dissecting type of disease, high American Society of anesthesiologists (ASA) status. While undertaking open surgical repair, preoperative cerebrospinal fluid drainage and using partial extracorporeal circulation has shown to reduce the risk of spinal and visceral ischemia.^87,89^ Although the risk of spinal cord ischemia was equal in two groups, the need for the spinal drain was lesser in the reintervention group.^90^ The aortic rupture is an independent predictor for treatment failure.^86^

### Future directions

The true incidence of PAU, clinical, and imaging behavior of both symptomatic and asymptomatic PAU is still to be determined. A broad consensus is required for surgical indications of PAU as well as predictors of its regression. Whether VV ruptures and progresses to IMH as primary or is predisposed by IMH as a secondary event needs to be seen. Conservative approach in cases of IMH needs to be defined by laid down criteria, as well as the sensitivity of fat-suppressed and nonfat suppressed MRI in precise detection of IMH needs to be documented. The prognostic significance of FID, IBP, and LIT needs to be ascertained in relation to PAU, IMH, and AD, respectively. The specificity of different imaging modalities to identify entry site tear needs to be established. Indication of endovascular intervention in cases of PAU and IMH coming into light by embolic events needs to be addressed. Stent-grafts covering the ulcer and part of IMH has shown promising initial results, which require confirmation by further studies.

In many instances, due to the presence of diffuse atherosclerotic changes, multiple PAU or long segment of IMH, stent-graft is required to cover a long segment of the aorta. The downside of this time-consuming procedure is the occurrence of spinal cord ischemia manifesting as leg weakness or non-recoverable paraplegia, further aggravated by renal insufficiency and associated peripheral arterial disease (PAD).^91^

Pua et al reported the use of a multilayer flow modulator (MFM) in one of the first such cases enabling the completion of a two-staged procedure into a single sitting.^92^ MFM promotes thrombosis of an aneurysm and at the same time maintains flow in side branches. The treatment lead to successful treatment with stabilization of aneurysm and no overt complications of side branch ischemia or endoleak at six months follow up imaging and clinical stability till two years post-procedure. There was only one technical limitation of the procedure requiring spacing the stent-grafts 5 cm apart, facilitating the function of MFM.

Short and midterm outcomes of TEVAR are encouraging; the long-term issue of the same, especially in regard to any complications, needs to be ascertained. There are several limitations encountered in the studies. In relation to PAU, there was an inability to accurately assess disease progression due to a lack of baseline study and lack of histopathological confirmation. In the case of AD, there was a wide variation in the appearance of intimal flap among different imaging modalities precluding accurate detection and categorization.

## Discussion and Conclusion

PAU is commonly encountered beyond the sixth decade of life with male predominance, albeit small in some instances. Hypertension, hyperlipidemia, and diabetes are widely associated comorbidities, with current or past smoking being the most common form of substance abuse. Symptomatic clinical presentation is variable among different studies, with positive cases having progressive nature. The patients frequently manifest background atherosclerotic burden in the form of involvement of cerebral-carotid, peripheral, or even renal circulation. PAU has a predilection for descending thoracic aorta and can be occasionally multiple. Prognosis of PAU depends on ulcer dimension and depth, whereas maximum thickness guides the same principle in cases of IMH. IMH closely follows PAU in distribution and localization. AD is complicated by either PAU or IMH, can also appear spontaneously after an episode of trauma. The presence of pleural or pericardial fluid indicates imminent or frank rupture. Hemopericardium signifies the same in disease localization to ascending aorta and can be life-threatening in cases of tamponade. Management in cases of thoracic aorta depends on the involved aortic segment. In cases of involvement of ascending aorta and or arch, the patient will invariably require urgent management, either open surgical or TEVAR, irrespective of symptomatology. In cases of descending thoracic involvement, medical management is apt in cases that are asymptomatic, those with stable IMH, pleural effusion, and or IBPs, as well as those with aortic diameter less than 50 mm and absence of hemopericardium.

PAU involving descending thoracic aorta required urgent management in a symptomatic patient with persistent or increasing pain, hemodynamically instability, those with expanding IMH, IBPS, and or hemothorax, as well as those with the appearance of hemopericardium. Emergency treatment is also required in cases showing large aortic diameter >60mm, saccular morphology of associated aneurysm, those with imminent or frank signs of rupture, and those showing fistulous communication with airway or esophagus. TEVAR is preferred in cases of infectious aortitis, cases showing signs of limb ischemia, and those with associated comorbidities precluding open surgical management.

In cases of abdominal aortic involvement, open surgical management is preferred in young patients as they usually are free from comorbidities and those with aortoenteric fistula as definite treatment. TEVAR in the abdominal aorta is preferred in those having embolic phenomenon or lower limb ischaemic events, cases of paravisceral PAU, and as bridging intermediate step in cases of aortoenteric fistulous communication.
